# Elucidating the Impact of Surfactants on the Performance of Dissolving Microneedle Array Patches

**DOI:** 10.1021/acs.molpharmaceut.1c00988

**Published:** 2022-03-02

**Authors:** Qonita Kurnia Anjani, Akmal Hidayat Bin Sabri, Emilia Utomo, Juan Domínguez-Robles, Ryan F. Donnelly

**Affiliations:** School of Pharmacy, Queen’s University Belfast, Medical Biology Centre, 97 Lisburn Road, Belfast BT9 7BL, U.K.

**Keywords:** dissolving microneedle array patches, surfactant, percentage of height reduction, drug delivery efficiency

## Abstract

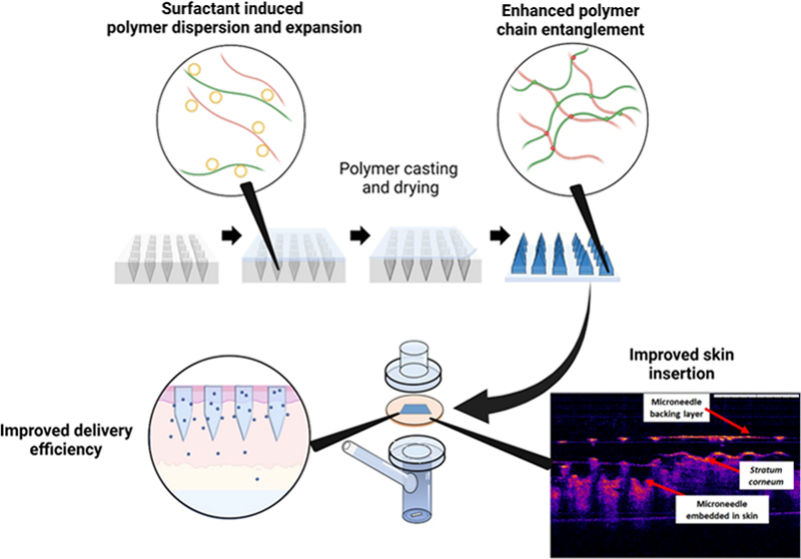

The need for biocompatible polymers capable of dissolving in the skin while exhibiting reasonable mechanical features and delivery efficiency limits the range of materials that could be utilized in fabricating dissolving microneedle array patches (MAPs). The incorporation of additives, such as surfactants, during microneedle fabrication might be an alternative solution to overcome the limited range of materials used in fabricating dissolving MAPs. However, there is a lacuna in the knowledge on the effect of surfactants on the manufacture and performance of dissolving MAPs. The current study explores the role of surfactants in the manufacture and performance of dissolving MAPs fabricated from poly(vinyl alcohol) (PVA) and poly(vinyl pyrrolidone) (PVP) loaded with the model drugs, ibuprofen sodium and itraconazole. Three nonionic surfactants, Lutrol F108, Pluronic F88, and Tween 80, in solutions at varying concentrations (0.5, 1.0, and 2.0% w/w) were loaded into these dissolving MAPs. It was discovered that all of the dissolving MAPs that incorporated surfactant displayed a lower reduction in the microneedle height (≈10%) relative to the control formulation (≈20%) when subjected to a compressive force of 32 N. In addition, the incorporation of surfactants in some instances enhanced the insertion profile of these polymeric MAPs when evaluated using *ex vivo* neonatal porcine skin. The incorporation of surfactant into ibuprofen sodium-loaded dissolving MAPs improved the insertion depth of MAPs from 400 μm down to 600 μm. However, such enhancement was not apparent when the MAPs were loaded with the model hydrophobic drug, itraconazole. Skin deposition studies highlighted that the incorporation of surfactant enhanced the delivery efficiency of both model drugs, ibuprofen sodium and itraconazole. The incorporation of surfactant enhanced the amount of ibuprofen sodium delivered from 60.61% up to ≈75% with a majority of the drug being delivered across the skin and into the receptor compartment. On the other hand, when surfactants were added into MAPs loaded with the model hydrophobic drug itraconazole, we observed enhancement in intradermal delivery efficiency from 20% up to 30%, although this did not improve the delivery of the drug across the skin. This work highlights that the addition of nonionic surfactant is an alternative formulation strategy worth exploring to improve the performance and delivery efficiency of dissolving MAPs.

## 1 Introduction

Being the largest organ in the human body, the skin functions as a barrier between the internal organs and the external environment. This multilayered organ consists of three distinctive histological layers: epidermis, dermis, and hypodermis.^1^ Despite being one of the most accessible organs, the efficient barrier function of the *stratum corneum* limits the range of therapeutics that are available for transdermal and intradermal delivery.^2^ For a drug molecule to traverse intact *stratum corneum*, it should ideally possess the following properties: Mw < 600 Da, Log *P*: 1.0–3.0, low melting point, hydrogen bonding group ≤ 2, nonirritating, and nonsensitizing.^3−5^ Due to these physiochemical requirements, the range of therapeutics that have been successfully delivered *via* conventional transdermal patches is limited. Given the limitations of the current transdermal patches, there is an impetus to explore alternative drug delivery strategies to expand and improve the range of molecules that can be delivered into and across the skin.

Described as a hybrid between the transdermal patch and hypodermic injection, microneedles are biomedical microdevices consisting of arrays of microprojections capable of breaching the *stratum corneum*.^6^ Upon application, microneedles generate transient microchannels within the skin that could be utilized as conduits for the intradermal and transdermal delivery of therapeutics. This drug delivery platform confers several advantages over conventional subcutaneous and intramuscular injection, such as painless drug administration and obviating first-pass hepatic metabolism.^7^ Due to the ease and simplicity of applying microneedle array patches (MAPs), this drug delivery system offers the opportunity for patient self-administration.^8^

Generally, microneedles can be classified into five distinct classes: solid, coated, hollow, hydrogel-forming, and dissolving MAPs.^9^ Dissolving MAPs involve encapsulating drug molecules within a polymeric matrix that forms the length of the microneedles. Upon application to the skin, the microneedles dissolve leading to drug release into the surrounding dermal tissues that ultimately traverses into the systemic circulation.^10^ In addition, the dissolution of the microneedle layer upon skin application is an innate self-disabling feature of dissolving microneedles that results in no biohazardous sharps post application. This could be of great advantage in mitigating the risk of needle stick injuries post application.^11^

Some of the materials that have been utilized in fabricating dissolving MAPs include sugars, such as maltose, sucrose, galactose, and trehalose.^12−14^ In addition, polymers, both natural and synthetic, have been explored as materials to manufacture dissolving MAPs. Some of the polymers that have been used in fabricating dissolving MAPs include poly(vinyl alcohol) (PVA),^15^ poly(methyl vinyl ether-*co*-maleic anhydride),^16^ poly(vinyl pyrrolidone) (PVP),^17^ carboxymethyl cellulose,^18^ hyaluronic acid,^19^ and silk fibroin.^20^ Despite these advantages, dissolvable polymers used in manufacturing dissolvable microneedles sometimes exhibit poor mechanical properties, especially when the microneedles are manufactured with a high drug loading.^21^ The need for biocompatible polymers capable of dissolving in the skin for drug delivery while exhibiting capability in resisting axial compression features limits the choice of materials that could be utilized in fabricating dissolving MAPs.

Various strategies have been employed to enhance the mechanical properties of dissolving MAPs. Increasing the polymer concentration in the manufacturing step is a potential solution.^22^ However, this strategy causes the manufacturing step to be more challenging due to the increased viscosity of the polymer blend, necessitating high centrifugation speed or very high positive pressure to fill the cavity of the microneedle molds in a consistent fashion.^18,23^ Alternatively, cross-linking the polymer used may improve the overall mechanical properties of the microneedles, but this approach may reduce the overall solubility of the polymer and in most instances change the type of microneedles from dissolving to hydrogel-forming that ultimately results in a completely different release profile.^24^ Another strategy is to incorporate additives along with the drug and polymer cast during microneedle fabrication. These additives may form strong interactions with the drug and polymer, resulting in improved mechanical properties.^25−27^ Some of the additives that have been explored include nanocomposites, such as gold nanocages^25^ and layered double-hydroxide nanohydroxides^26^ that form strong interaction with the polymer matrix, resulting in enhanced microneedle strength. This is akin to the “brick-and-mortar” structure where the nanostructure forms the brick while the polymer matrix is represented by the mortar.^28^ The incorporation of these nanostructures within the polymeric matrix of microneedles has been explored as a preventative strategy to mitigate the reduction in the mechanical properties of dissolving MAPs upon drug addition.

Alternatively, incorporating surfactants has been explored as a strategy, albeit less popular, to improve the fabrication process and overall performance of dissolving MAPs.^29−31^ With this approach, the surfactant used functions mostly as an external plasticizer to reduce the overall rigidity and brittleness of the dissolving MAPs upon drying. Doing so enables the ease of demolding without fracturing the microneedle tips.^32^ In addition, the use of surfactants in the manufacture of microneedles has been widely explored in the manufacture of coated microneedles.^33−37^ In this instance, surfactants help to reduce the surface tension of coating solutions to ensure improved and consistent wetting of the microneedle surface.^38,39^ Nevertheless, the role and effect of surfactants in the manufacture and performance of dissolving microneedles are yet to be fully explored.

Indeed, the inclusion of surfactants that behave as a wetting agent may aid the contact of the dermal interstitial fluid with the length of dissolving microneedles, which are fabricated from a mixture of water-soluble polymers. Upon puncturing the *stratum corneum*, the dissolving microneedles are implanted into the highly aqueous dermis. Here, the polymeric microneedles come into contact with the interstitial fluid and begin to bind with water molecules that hydrate the hydrophilic pendant groups on the polymers, leading to formation of primary bound water layers. The formation of these primary bound water layers then leads to polymer swelling, which exposes the hydrophobic region of the polymer (the backbone) and which also interacts with water molecules *via* Van der Waals forces, forming the secondary bound water layers and leading to further swelling. The presence of nonionic surfactants in this instance aids the ingress of water molecules and the formation of water-binding layers within the polymeric microneedle matrix. This ultimately causes additional water molecules to imbibe into the needle structures due to osmosis leading to rapid polymer swelling and dissolution culminating in the release of drug molecules into the surrounding dermal tissues.^40^ Therefore, the inclusion of surfactants into the microneedles may provide a strategy to improve the wettability of the polymeric microneedle tips, which would in turn enhance microneedle dissolution leading to improved delivery efficiency.

Hence, the current work aims to investigate the role of surfactant in the manufacture and performance of dissolving MAPs fabricated from poly(vinyl alcohol) (PVA) and poly(vinyl pyrrolidone) (PVP). Three regulatory-approved nonionic surfactants, Lutrol F108, Pluronic F88, and Tween 80, at three different concentrations (0.5, 1.0, and 2.0% w/w) were incorporated into the dissolving MAP formulations loaded with two model compounds, ibuprofen sodium and itraconazole. Nonionic surfactants were selected for this work, as it has been reported that nonionic surfactants display a better safety profile, resulting in less cutaneous irritation in comparison to ionic surfactants.^41^ A series of experiments were conducted to characterize the appearance, mechanical properties, insertion profile, drug loading, and drug delivery efficiency of the formulations developed. It is hoped that the current work provides fundamental insight into the useful role of surfactants in improving the properties and performance of dissolving MAPs.

## 2 Experimental Section

### 2.1 Materials

Ibuprofen sodium salt and poly(vinyl alcohol) 9–10 kDa were purchased from Sigma-Aldrich (Dorset, U.K.). Itraconazole (purity, 98%) and Tween 80 were purchased from Tokyo Chemical Industry (Oxford, U.K.). Lutrol F108 and Pluronic F88 Pastille were provided by BASF (Ludwigshafen, Germany). Poly(vinyl pyrrolidone) (PVP) 58 kDa and PVP 90 kDa were provided by Ashland (Kidderminster, U.K.). Ultrapure water was obtained from a water purification system (Elga PURELAB DV 25, Veolia Water Systems, Dublin, Ireland). All other chemicals and materials were of analytical grade and purchased from Sigma-Aldrich (Dorset, U.K.) or Fisher Scientific (Loughborough, U.K.). Full-thickness neonatal porcine skin was obtained from stillborn piglets in less than 24 h *post-mortem* and stored at −20 °C until use.

### 2.2 Determination of the Contact Angle of Surfactant Solutions with Ibuprofen Sodium and Itraconazole

To observe the contact angle of each surfactant solution with the surface of ibuprofen sodium and itraconazole, an Attension Theta optical tensiometer (Biolin Scientific, Gothenburg, Sweden) with the sessile drop method was used. Initially, 50 mg of each drug was weighed and compressed into tablets using a 4 tonne compression force to obtain drug tablets with a flat surface. Surfactant solutions were prepared in concentrations of 0.5% w/w, 1.0% w/w, and 2.0% w/w in all cases of Tween 80, Lutrol F108, and Pluronic F88. Moreover, water was used as the control. A volume of 4 μL of each surfactant solution was dropped onto the surface of the drug tablet. The contact angle was measured at 30.24 s after the release of the droplet. Subsequently, the results were analyzed using OneAttension software. This experiment was performed in triplicate.

### 2.3 Determination of Size and Polydispersity Index (PDI) of Drugs Loaded in Surfactant Solution

A 2 mg aliquot of each drug, ibuprofen sodium salt or itraconazole, was dispersed into an Eppendorf tube containing 4 mL of aqueous surfactant solution using a vortex at 2500 rpm for 1 min. Following this, the aqueous mixture was transferred into a plastic disposable cell (length, 12 mm; height, 45 mm; width, 12 mm) prior to analysis. In this study, particle size distribution and polydispersity index were determined by dynamic light scattering (DLS) using a NanoBrook Omni analyzer (Brookhaven, New York). The analysis was performed at 25 °C with 3 min equilibration time. Results were obtained from three replicate measurements.

### 2.4 Fabrication of Dissolving MAPs

**Figure 1:**
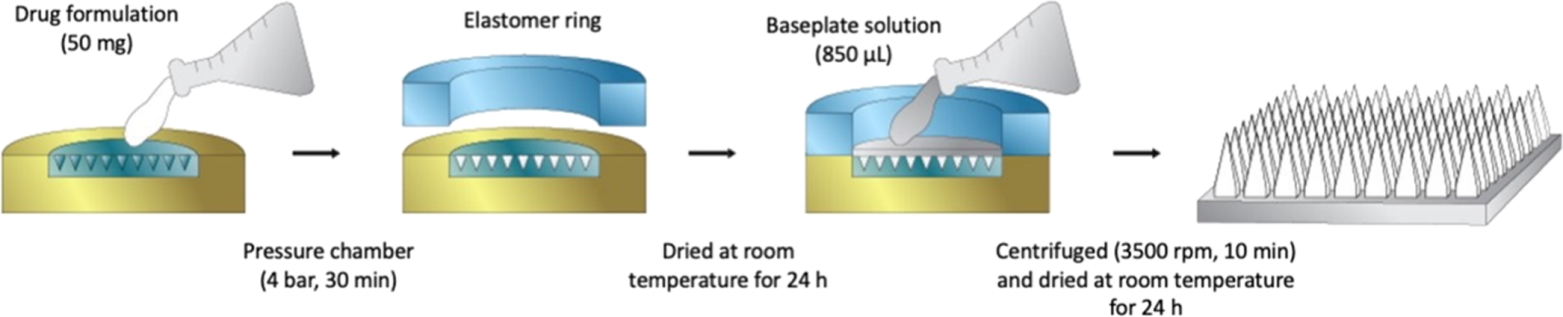
Schematic representation of dissolving MAP fabrication.

Dissolving MAPs were prepared using a double casting method, as described previously,^21,42^ with a slight modification. A polymeric solution of 20% w/w of PVA (9–10 kDa) and 20% w/w of PVP (58 kDa) was used as the matrix for the needle tips, whereas a polymeric solution of 30% w/w of PVP (90 kDa) and 1.5% w/w of glycerol was used as the baseplate layer of the patches. Briefly, approximately 50 mg of each drug-containing mixture (outlined in Table 1 and illustrated in Figure 1) was poured into a silicone mold (16 × 16 pyramidal needle density, 850 μm height, 300 μm width at base, 300 μm interspacing, and 0.36 cm^2^ patch area) as a first layer. The molds were placed in a positive pressure chamber at 4 bar for 5 min. The excess formulations of the first layer were then removed using a spatula and the molds were dried for 30 min inside the positive pressure chamber at 4 bars. Afterward, elastomer rings (external diameter 23 mm, internal diameter 18 mm, thickness 3 mm) were attached on top of the molds using a glue solution prepared from an aqueous blend of 40% w/w of PVA (9–10 kDa). After drying at room temperature for 6 h, 850 μL of the second layer, an aqueous blend of 30% w/w of PVP (90 kDa) and 1.5% w/w of glycerol, was poured into the molds, which were then centrifuged at 3500 rpm for 10 min. The molds were then dried at room temperature for 24 h, and the sidewalls formed were removed using scissors and then further dried at 37 °C for 12 h. Finally, there were 10 MAP formulations for each drug, ibuprofen sodium and itraconazole (Table 2).

**Table 1:** Formulation for Dissolving MAP Preparation

drug	components (%w/w)
ibuprofen sodium	30% drug, 20% w/w of aqueous polymer blend (PVA (9–10 kDa) and PVP (58 kDa)), 50% surfactant solution
ibuprofen sodium (control)	30% drug, 20% w/w of aqueous polymer blend (PVA (9–10 kDa) and PVP (58 kDa)), 50% deionized water
itraconazole	15% drug, 45% w/w of aqueous polymer blend (PVA (9–10 kDa) and PVP (58 kDa)), 40% surfactant solution
itraconazole (control)	15% drug, 45% w/w of aqueous polymer blend (PVA (9–10 kDa) and PVP (58 kDa)), 40% deionized water

**Table 2:** Formulation Codes Based on the Concentration of Surfactant in the Surfactant Solution

	concentration of surfactant in the surfactant solution (%w/v)		
MAP formulation code	Pluronic F88	Lutrol 108	Tween 80
control			
P0.5	0.5		
P1	1.0		
P2	2.0		
L0.5		0.5	
L1		1.0	
L2		2.0	
T0.5			0.5
T1			1.0
T2			2.0

### 2.5 Characterization of Dissolving MAPs

Morphology of dissolving MAPs was visualized using a digital light microscope (Leica EZ4 D, Leica Microsystems, Milton Keynes, U.K.). Differential scanning calorimetry (DSC) analysis of pure drugs and MAP formulations was performed using a DSC Q100 (TA Instruments, Elstree, Hertfordshire, U.K.).

### 2.6 Evaluation of Mechanical Resistance of Dissolving MAPs

Mechanical resistance of dissolving MAPs was evaluated using a TA-TX2 Texture Analyzer (TA) (Stable Microsystems, Haslemere, U.K.) in compression mode, as previously reported.^43,44^ The height of needles before and after pressure application was measured and recorded using the digital light microscope. The percentage needle height reduction was then calculated using eq 1
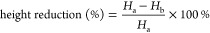
1where *H*_a_ is the height before compression and *H*_b_ is the height after compression.

### 2.7 Insertion Properties of Dissolving MAPs

The insertion and penetration depth of dissolving MAPs were determined using an EX-101 optical coherence tomography (OCT) microscope (Michelson Diagnostics Ltd., Kent, U.K.), as reported previously,^45^ following insertion into full-thickness neonatal porcine skin and Parafilm M. ImageJ (National Institutes of Health, Bethesda, MD) was used to measure the height of needles inserted.^46^ The change in needle depth within the skin due to dissolution was monitored using OCT over a period of 1 h.

### 2.8 Determination of Drug Content in the Needles

A MAP was placed in 4 mL of deionized water and sonicated for 30 min to dissolve the hydrophilic polymer. The mixture was added to 4 mL of deionized water (for ibuprofen sodium-containing MAPs) and acetonitrile (itraconazole-containing MAPs) and sonicated for 30 min. The mixture was centrifuged at 14,500 rpm for 15 min prior to high-performance liquid chromatography (HPLC) analysis.

### 2.9 *In Situ* Dissolution Study

**Figure 2:**
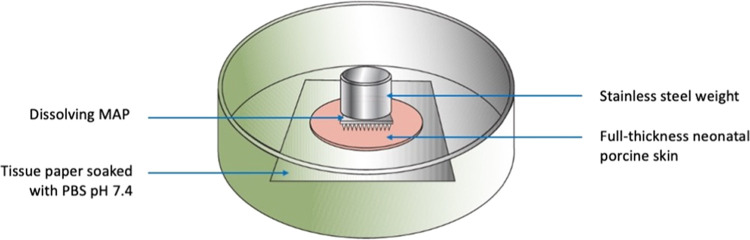
Schematic illustration of *in situ* dissolution study for dissolving MAPs using excised full-thickness neonatal porcine skin.

Prior to the experiment, the skin was pre-equilibrated in phosphate buffer saline (PBS) (pH 7.4) for 30 min. *In situ* MAP dissolution in the excised full-thickness neonatal porcine skin was assessed for the selected MAP formulation over a period of 1 h following MAP insertion using manual thumb pressure.^21^ A 5.0 g cylindrical stainless steel weight was placed atop the MAPs (Figure 2) to avoid MAP expulsion during the study. The plate was closed and stored at 37 °C for 1 h. The morphology of MAPs was then observed under the digital microscope following MAP detachment from the skin.

### 2.10 *Ex Vivo* Skin Deposition Study

**Figure 3:**
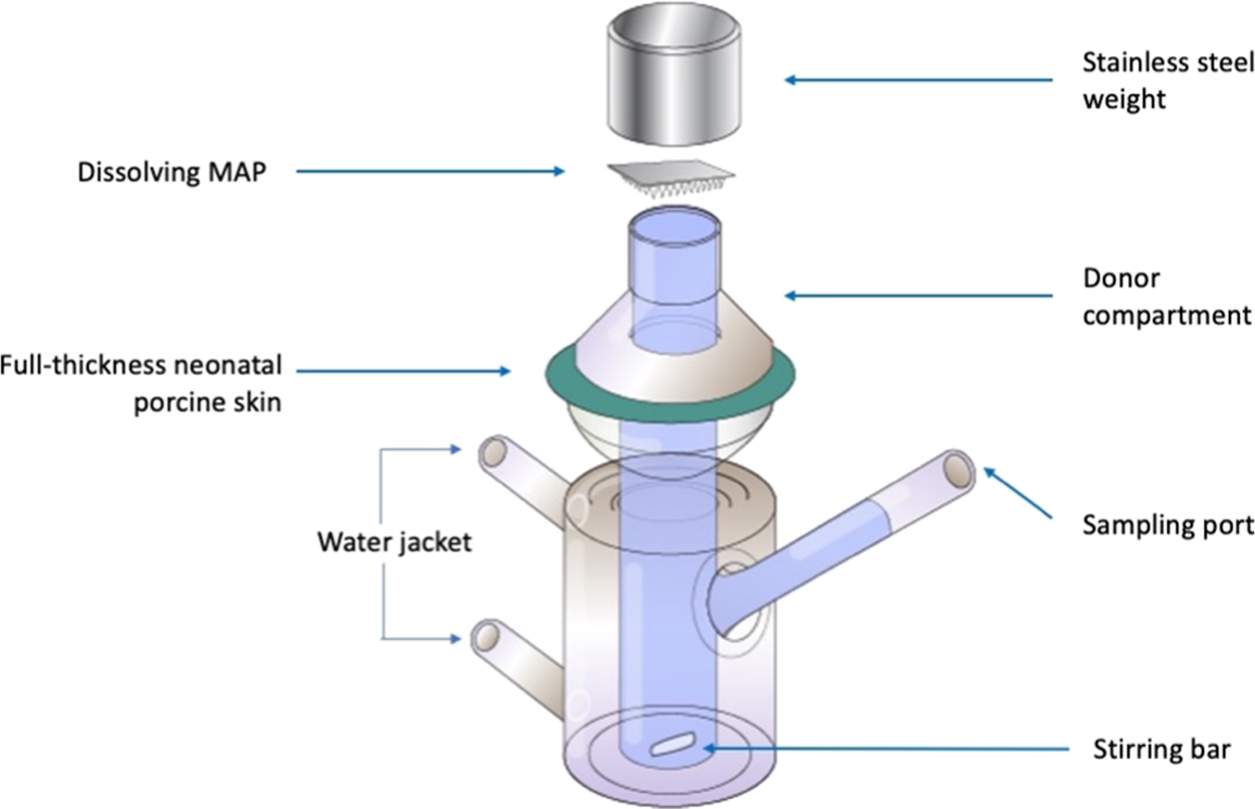
Schematic illustration of the modified Franz cell setup for *ex vivo* skin deposition studies of dissolving MAPs using full-thickness neonatal porcine skin.

A modified Franz cell (Permergear, Hellertown, PA) setup was adapted to evaluate the drug deposition in the skin and drug permeation to the receptor compartment over 24 h (Figure 3). Briefly, excised full-thickness neonatal porcine skin (diameter 28 mm) was attached to the donor compartment of Franz cells using cyanoacrylate glue. Dissolving MAPs were inserted into the skin using manual thumb pressure applied for 20 s. The donor compartment was then attached to the receptor compartment containing PBS (pH 7.4). A 5.0 g cylindrical stainless steel weight was placed atop the MAPs. The Franz cells were stirred at 600 rpm, and the temperature was maintained at 37 ± 1 °C. At 24 h, the skin from all donor compartments was detached and PBS from the receptor compartment was collected for further analysis.

To extract the drug from the skin sample, 0.5 mL of deionized water was added to samples and homogenized at 50 Hz using a Tissue Lyser (Qiagen Ltd., Manchester, U.K.) for 15 min. The sample was added with 1 mL of methanol (for ibuprofen sodium sample) and acetonitrile (itraconazole sample) and rehomogenized at 50 Hz using the Tissue Lyser for 15 min. All samples were sonicated for 1 h and filtered using 0.2 μm nylon membranes prior to HPLC injection. To extract the drug from the receptor compartment, 8 mL of appropriate solvent was added to the PBS and sonicated for 1 h. The samples were then centrifuged at 14,500 rpm for 15 min, and the supernatant was collected for HPLC analysis.

### 2.11 Instrumentation and Chromatographic Condition for the Analytical Method

Drug quantification in this work was performed using HPLC (Agilent Technologies 1220 Infinity UK Ltd, Stockport, U.K.). Analysis of the drugs was carried out individually using a Spherisorb ODS1 column (150 mm × 4.6 mm internal diameter, 5 μm particle size) (Waters, Ireland), with a flow rate of 1 mL/min, and at ambient temperature. The mobile phase, injection volume, and UV detector used for each drug are presented in Table 3. The chromatograms were analyzed using the Agilent ChemStation Software B.02.01. The International Council of Harmonisation (ICH) 2005 guidelines were followed as a reference to assess both analytical methods.

**Table 3:** Parameters of HPLC Analysis for Ibuprofen Sodium and Itraconazole Quantification

analyte	mobile phase	injection volume (μL)	UV detection (nm)
ibuprofen sodium	water (with triethylamine 0.05%, pH 8.0 adjusted using phosphoric acid): acetonitrile (75:25 v/v)	50	263
itraconazole	water (with triethylamine 0.05%, pH 8.0 adjusted using phosphoric acid): acetonitrile (28:72 v/v)	20	203

### 2.12 Statistical Analysis

Statistical analysis was performed using GraphPad Prism version 8.0 (GraphPad Software, San Diego, California). All experimental results were presented as means ± standard deviation (SD) unless otherwise stated. One-way analysis of variance (ANOVA) was used for the comparison of multiple cohorts. In all cases, *p* < 0.05 was used to denote statistically significant, where *p*-value outputs were 0.033(*), 0.002(**), <0.001(***), and <0.0001 (****).

## 3 Results and Discussion

### 3.1 Determination of the Contact Angle of Surfactant Solutions with Ibuprofen Sodium and Itraconazole

**Figure 4:**
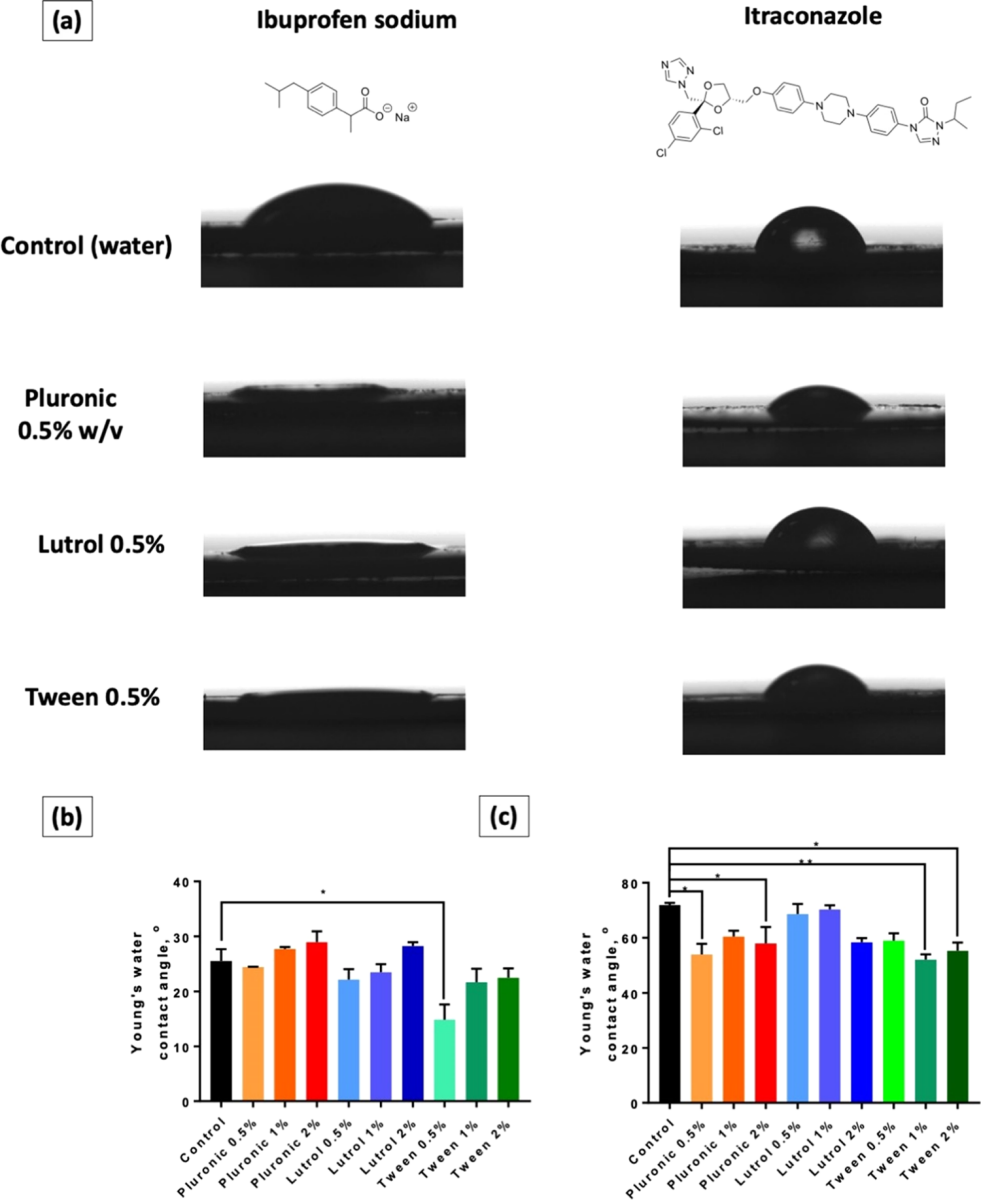
(a) Representative images for water contact angle measurement for pure drug solution along with drug solution with different polymeric surfactants at 0.5% w/w. (b) Water contact angle measurement for ibuprofen sodium drug solution with different polymeric surfactant concentrations. (c) Water contact angle measurement for itraconazole drug solution with different polymeric surfactant concentrations. Data are expressed as means + standard error of the mean (SEM), *n* = 3. Differences were calculated using one-way ANOVA, followed by Dunnett’s multiple comparison post hoc test with the pure drug solution set as a control and deemed significant at *p* < 0.05.

This part of the work was carried out to assess the effect of surfactants on the wettability properties of the drugs. The concentrations of surfactants used were 0.5, 1.0, and 2.0% (w/w). These concentrations were selected as these were the typical concentrations of surfactants used in the manufacture of polymeric microneedles.^10,17^ In addition, the use of a high concentration of surfactant would not only increase the propensity of inducing skin irritation but would also result in incomplete drying of MN films as shown by Cárcamo-Martínez et al.^35^ In Figure 4a, it can be seen that the contact angles of surfactant solutions were lower compared to that of water, indicating the ability of surfactants to decrease the surface tension, resulting in lower contact angles. In the case of ibuprofen sodium, the graph in Figure 4b shows that only Tween 80 0.5% (w/v) solution exhibited a significant difference compared to the control (*p* < 0.05). However, all other solutions possessed a contact angle below 30°, including the control. This indicated that ibuprofen sodium was considered a hydrophilic compound with a good wettability property.^47^ Accordingly, the presence of surfactant solutions did not affect the overall hydrophilicity of this drug. On the contrary, the effect of surfactants on the wettability of itraconazole was far more significant. Due to the hydrophobicity of itraconazole, a solid–vapor interface is formed upon contact with water. Accordingly, the surfactant was able to adsorb onto the surface of the drug particle, resulting in better wettability properties.^48^ This was proven by the results presented in Figure 4c. Tween 80 and Pluronic F88 showed a significant reduction in the water contact angle on the itraconazole surface. On the other hand, despite the nonsignificant difference compared to the control, Lutrol F108 was still able to lower the contact angle on the drug tablet. In all cases, no significant difference was observed among all concentrations of surfactant investigated. This phenomenon can be explained by the relation between the contact angle and the critical micelle concentration (CMC) value of the surfactant. With regard to Tween 80, it has been previously reported that its CMC value is 0.0019%.^49^ In this study, the concentrations used were higher than the CMC value. Accordingly, the measured contact angle was comparably similar. On the other hand, the CMC value of Pluronic F88 is not measurable at room temperature as it needs to reach the critical micelle temperature (CMT) to form micelles. As a result, no meaningful reduction was observed in the contact angle.^50^ The other surfactant, Lutrol F108, was reported for its CMC value of 4.5%.^51,52^ As all of the concentrations used were below the CMC value, thus, it was clearly observed that the solution of Lutrol F108 2% (w/w) possessed a lower contact angle compared to the other concentrations. Overall, these results indicated that the addition of surfactants to formulate poorly water-soluble drugs (i.e., itraconazole) was able to successfully increase the wettability of the drug. However, in the case of water-soluble drugs (i.e., ibuprofen sodium), there was no significant effect shown following the addition of surfactants.

### 3.2 Effect of Surfactant on the Particle Size of Ibuprofen Sodium and Itraconazole

**Figure 5:**
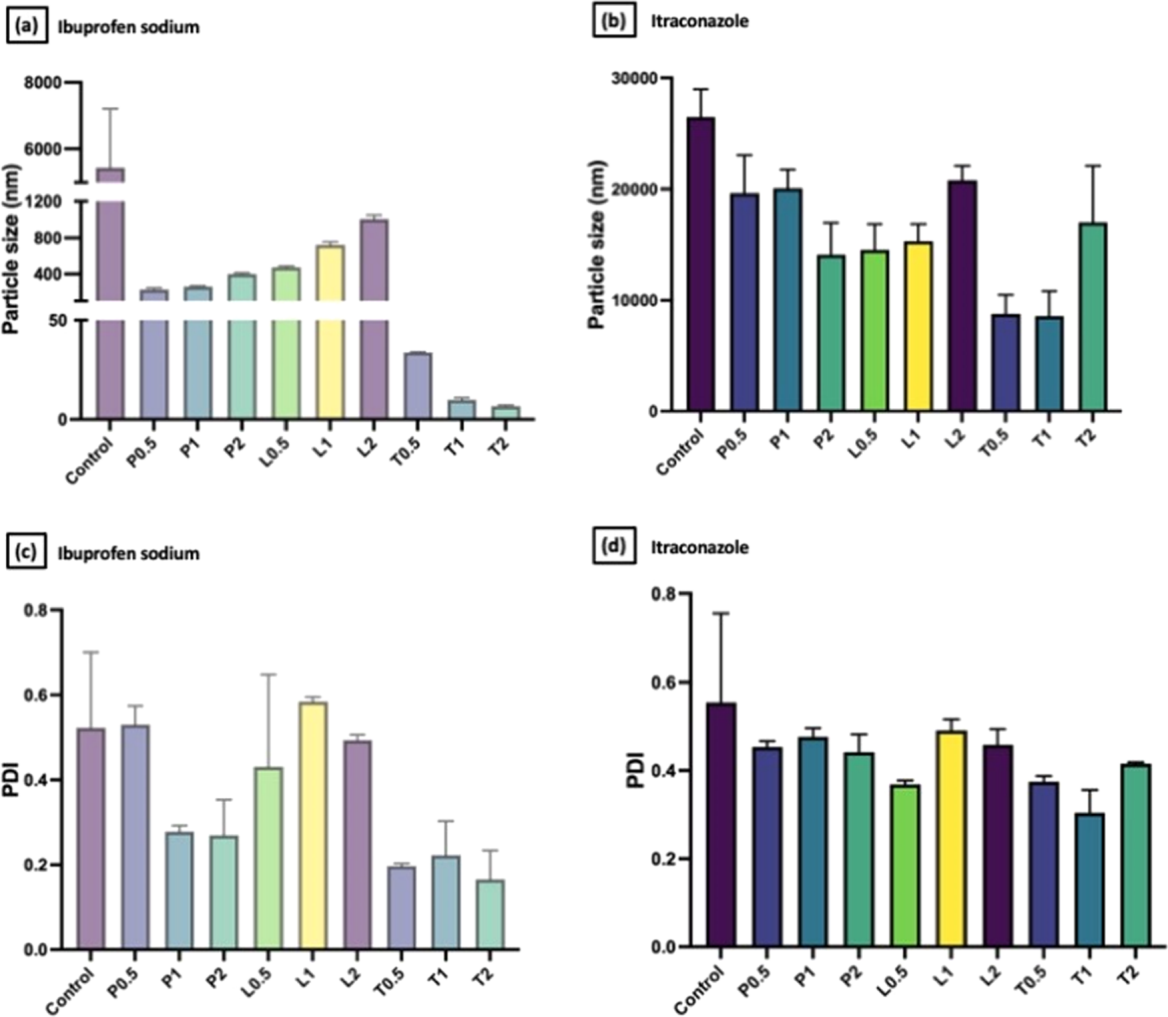
Comparison of the particle size distribution of (a) ibuprofen sodium and (b) itraconazole dispersed in different surfactant solutions with varied concentrations (means + SD, *n* = 3). Comparison of polydispersity index of (c) ibuprofen sodium and (d) itraconazole dispersed in different surfactant solutions with varied concentrations (means + SD, *n* = 3).

Prior to microneedle fabrication, dynamic light scattering was conducted on the drug suspension in the presence and absence of different surfactants of varying concentrations. The results (Figure 5) obtained were in good agreement with the contact angle experiment results as the addition of surfactant indeed had an impact on the overall reduction of drug particle size upon mixing. With regard to ibuprofen sodium, although it is categorized as a water-soluble compound, however, the amount of liquid used in this part of the experiment was not adequate to dissolve all of the provided drugs, resulting in the ability to measure the drug particle size.^53^ Accordingly, the reduction of drug particle size was well pronounced upon the addition of surfactants. The addition of Pluronic F88 and Lutrol F108 resulted in at least a 10-fold reduction in drug particle size, while the addition of Tween 80 solution at concentrations of 1 and 2% w/w resulted in around a 500-fold decrease in the particle size of ibuprofen sodium. On the other hand, it was also clear that the addition of surfactant did result in a reduction in drug particle size for the poorly water-soluble drug, itraconazole, upon mixing. However, the reduction in particle size was less effective when compared to that of ibuprofen sodium. Nevertheless, it was apparent that Tween 80 still resulted in the greatest reduction in drug particle size relative to Pluronic F88 and Lutrol F108 for both ibuprofen sodium and itraconazole. The reduction in drug particle size might be related to the drug solubility in water. In general, surfactants were able to decrease the surface tension of water. This resulted in the increase of drug solubility followed by the average reduction in the overall drug particle size. Additionally, as mentioned in the previous section it was clear that the huge reduction of particle size in both drugs after the addition of Tween 80 was because the concentrations used were above its CMC value. Under this condition, micelles were formed resulting in the more effective solubilization of drug particles.^54^ In contrast, as the concentration of Pluronic F88 and Lutrol F108 was below the CMC value, the solubilization was found to be lower than that of Tween 80 resulting in less reduction in the overall drug particle size.

### 3.3 Fabrication and Characterization of Dissolving MAPs

**Figure 6:**
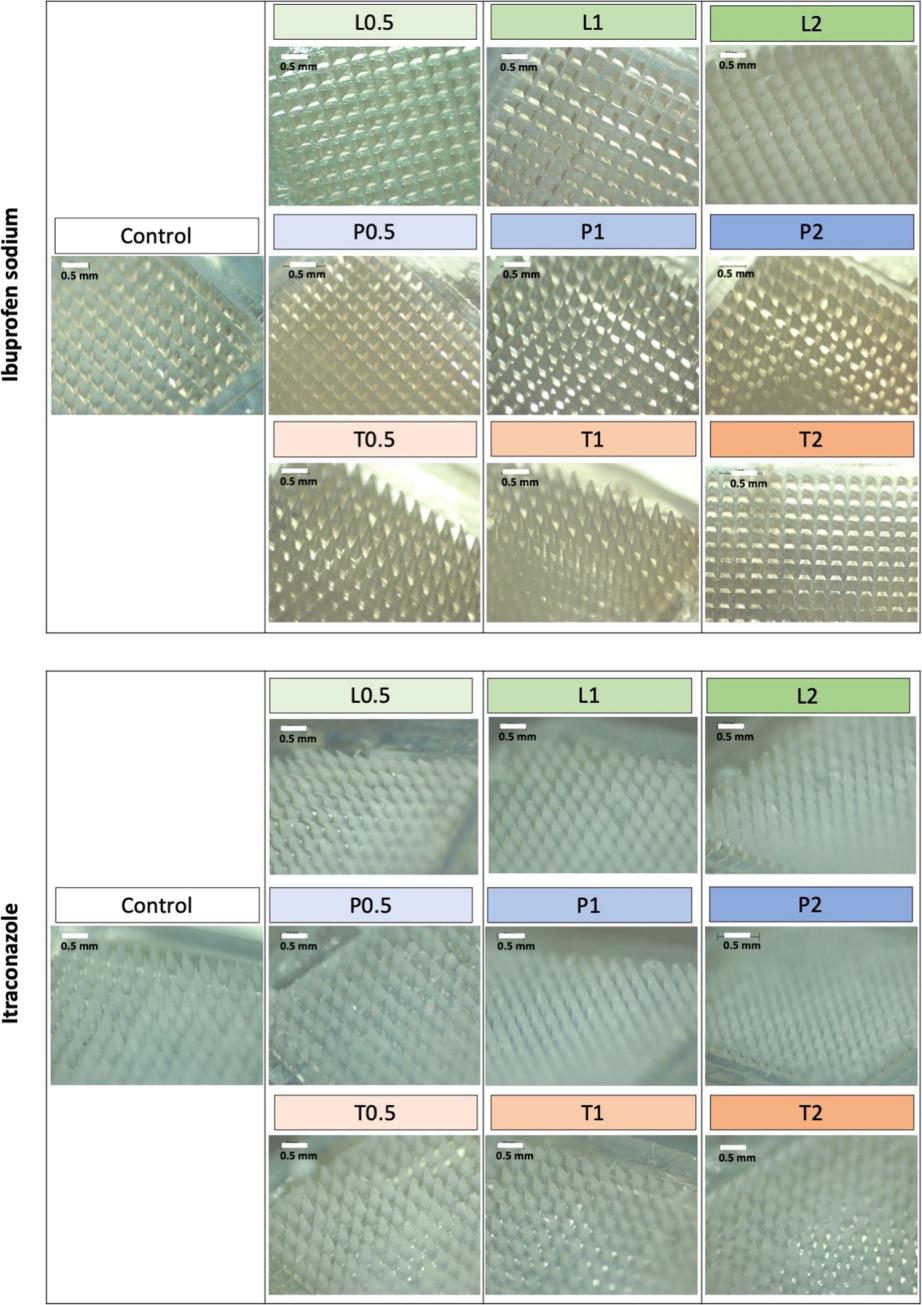
Digital images of dissolving MAPs composed of a pyramidal shape, 850 μm height, 300 μm width at base, and 300 μm interspacing.

Upon characterizing the drug particle size in the absence and presence of surfactants, dissolving MAP formulations of varying surfactant concentrations were fabricated *via* polymer casting and micromolding. The resulting polymeric MAPs are shown in Figure 6. Visual inspection *via* microscopy shows that all of the patches display visible microprojections. The MAPs manufactured from the polymeric blend of PVP and PVA loaded with ibuprofen sodium appeared clear, while those loaded with itraconazole appeared off-white in physical appearance.

**Figure 7:**
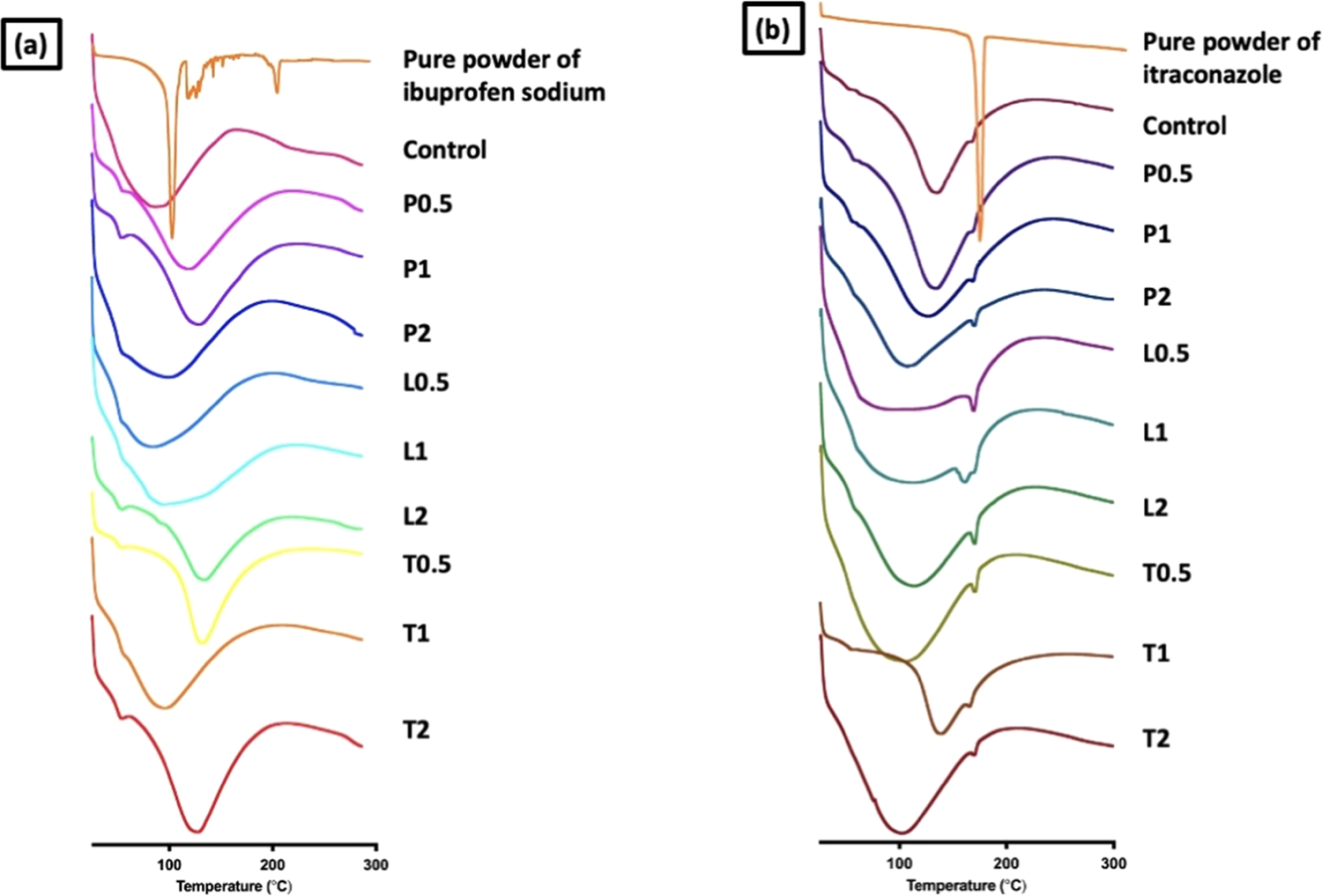
DSC analysis of dissolving MAPs containing (a) ibuprofen sodium and (b) itraconazole.

To gauge the solid state of the drug molecule that has been loaded into the polymeric MAPs, X-ray powder diffraction (XRPD) and DSC were performed, and the results are displayed in Figure 7. DSC thermal analysis was performed to evaluate the physicochemical interaction between the surfactant, polymer, and drug and determine whether drug crystallinity was affected during the MAP fabrication step. The thermogram for pure ibuprofen sodium powders revealed sharp endotherms at 103 °C, representing the melting point for the drug. However, upon incorporating into MAP formulations, there was no discernible endotherm from the DSC thermogram, which suggests that ibuprofen sodium is present in a state of low crystallinity within the MAP formulations. On the other hand, the thermograms for pure itraconazole powders revealed sharp endotherms at 163 °C, representing the melting point for the drug. When loaded into the microneedle formulation, the endotherm peak at 163 °C was much smaller. This may also suggest that itraconazole was in a state of low degree of crystallinity within the MAP formulations. However, such reduction in the itraconazole endotherm at 163 °C might also be attributed to simple dilution when the drug was loaded into dissolving MAPs.^55^

### 3.4 Mechanical Resistance of MAPs

**Figure 8:**
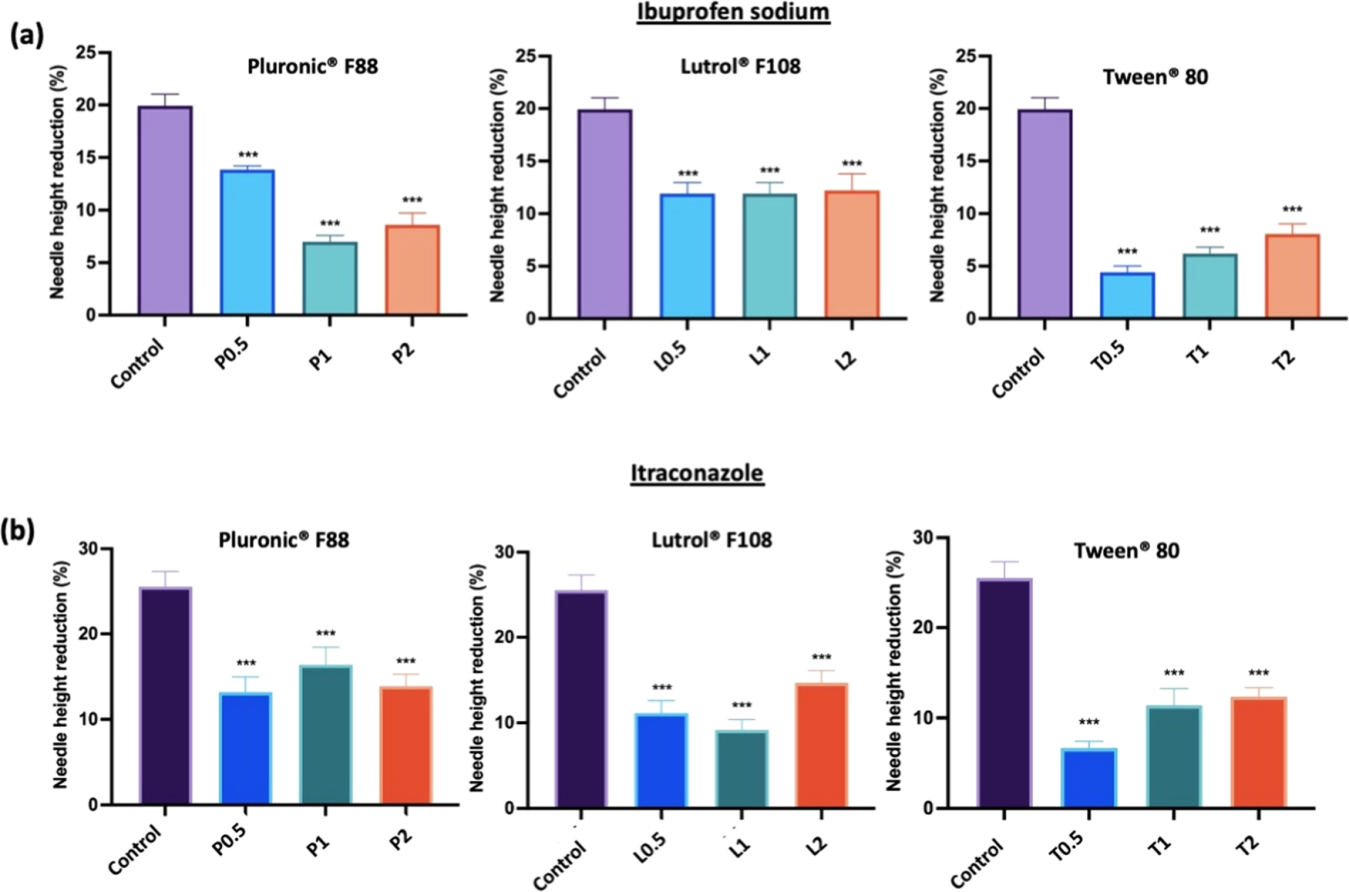
Comparison of height reduction percentage of needles loaded with (a) ibuprofen sodium and (b) itraconazole following application of a force of 32 N using TA (means + SD, *n* = 20). Differences were calculated using one-way ANOVA, followed by Dunnett’s multiple comparison post hoc test with the pure drug solution set as a control and deemed significant at *p* < 0.05.

It can be seen from Figure 8a,b that the incorporation of surfactants resulted in an improvement in the mechanical strength of the dissolving MAPs fabricated. In the case of ibuprofen sodium-loaded dissolving MAPs, the incorporation of surfactants for all three concentrations evaluated resulted in an increase in the mechanical resistance of the MAPs as evidenced from the decrease in the percentage of needle height reduction upon being subjected to compressive force. On the other hand, the incorporation of surfactants (Lutrol F108 and Tween 80) into itraconazole-loaded dissolving MAPs also significantly enhanced the overall mechanical resistance of the fabricated MAP (*p* < 0.05). Overall, the incorporation of surfactant enhanced the mechanical resistance of dissolving MAPs. Such an observation may be attributed to molecular interaction between the surfactant molecules and polymers used in fabricating the microneedle matrix.

It can be seen that the enhancement in the mechanical properties of the dissolving microneedles, as shown in Figure 8, can be attributed to the additive used during the microneedle manufacturing stage. In the case of all dissolving polymeric microneedles, the addition of nonionic surfactants conferred a significant (*p* < 0.05) degree of resistance against the compressive force typically encountered during skin insertion. The addition of these surfactants into the microneedles may provide some degree of plasticization to the overall microneedle matrix.^56^ Such plasticization mitigated microneedle height reduction upon compression. The addition of these surfactants falls under the category of external plasticization and can be carried out directly during the preparation of the aqueous polymer blend prior to micromolding.^57^ During the preparation of the polymer blend, the PVP and PVA chains open up enabling the surfactant to enter and interact with the hydrogen bonding groups on PVP and PVA. Hydrogen bonding occurs between the hydroxyl groups of the surfactant and the hydroxyls group of PVA. In addition, the pyrrolidone group present on PVP also acts as a good proton acceptor, which facilitates hydrogen bonding with the hydroxyl groups on the surfactant.^58^ This interaction reduces the intramolecular rigidity between the PVP and PVA polymers conferring some degree of fracture resistance upon compression. In addition, there is a possibility for PVP and PVA used in fabricating the microneedle matrix to form polymer–polymer interactions with the nonionic polymeric surfactant used *via* chain entanglement within the microneedle. Such supramolecular interactions between polymers have been shown by Lamm et al., and they significantly improve the tensile strength of polymers resulting in stronger microneedles capable of resisting the compressive force typically encountered during skin insertion.^59,60^

### 3.5 Microneedle Insertion Studies

**Figure 9:**
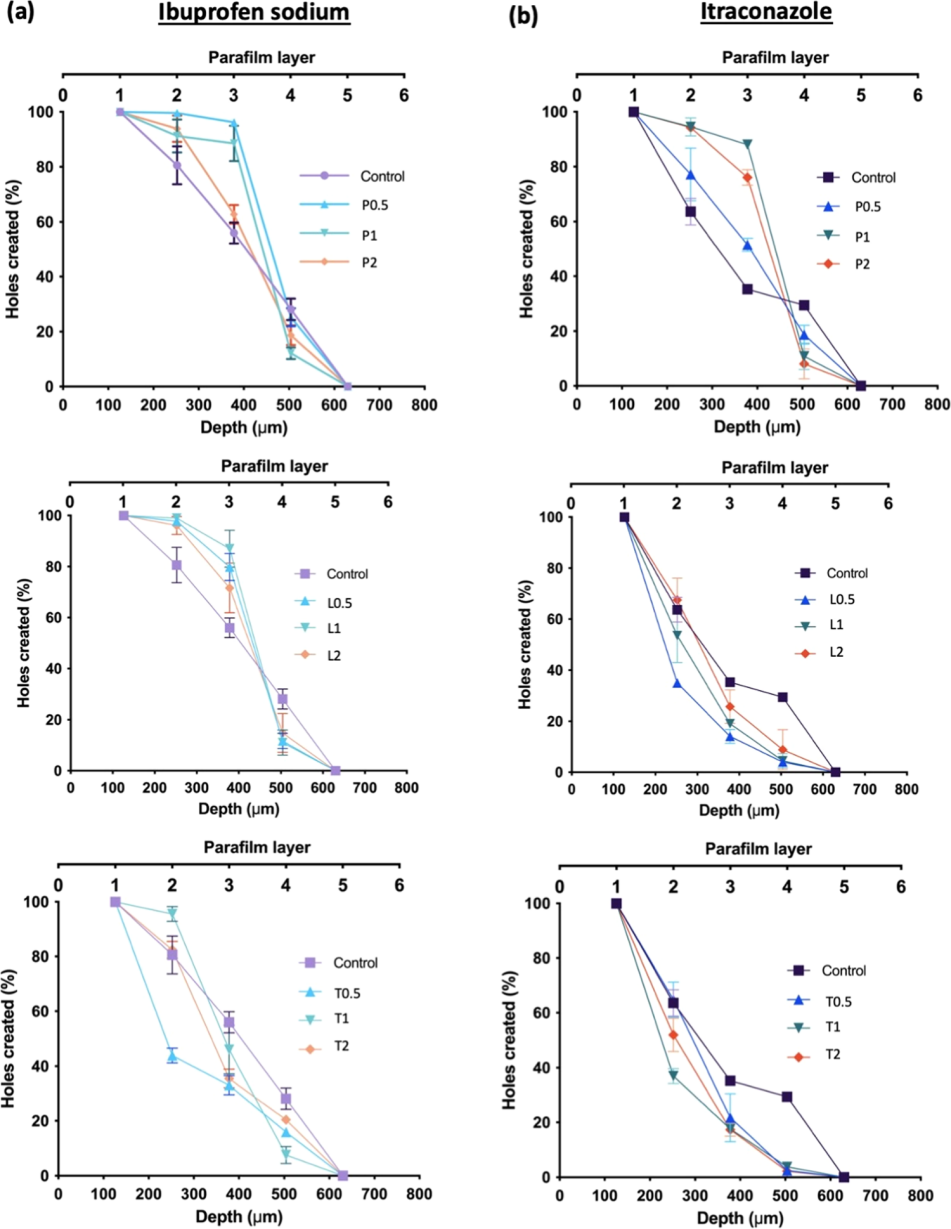
Percentage of holes created in each Parafilm M layer and estimated insertion depths following insertion of dissolving MAP formulations loaded with (a) ibuprofen sodium and (c) itraconazole (means ± SD, *n* = 3).

From Figure 9, it can be seen that all of the MAP formulations fabricated were capable of breaching the first layer of Parafilm with 100% insertion upon application. With respect to ibuprofen sodium-loaded dissolving MAPs, it can be seen that the incorporation of Pluronic F88 and Lutrol F108, for all formulations evaluated, resulted in MAPs with better insertion efficiency per layer as a function of the Parafilm layer number, with the deepest layer penetrated by the microneedle patch being the fourth layer. In contrast, the incorporation of Tween 80 into the formulation resulted in poor insertion efficiency at a low surfactant concentration (0.5% w/v of surfactant solution). However, when the concentration of surfactant was increased (1.0–2.0% w/v of surfactant solution), the insertion profiles were relatively similar to that of the control MAP formulation that was devoid of surfactant. Overall, this data suggests that the incorporation of Pluronic F88 and Lutrol F108 improved the insertion profile for ibuprofen sodium-loaded dissolving MAPs. In contrast, the incorporation of Tween 80 did not confer any improvement in the insertion profile for ibuprofen sodium-loaded dissolving MAP formulations. On the other hand, it can be observed that for itraconazole-loaded MAP formulations, a completely different trend was observed. The addition of Lutrol F108 and Tween 80, for all concentrations evaluated, resulted in MAPs with poor insertion efficiency as a function of the Parafilm layer number. In contrast, the addition of Pluronic F88 did, however, improve the microneedle insertion profile within the first three Parafilm layers relative to the control formulation.

**Figure 10:**
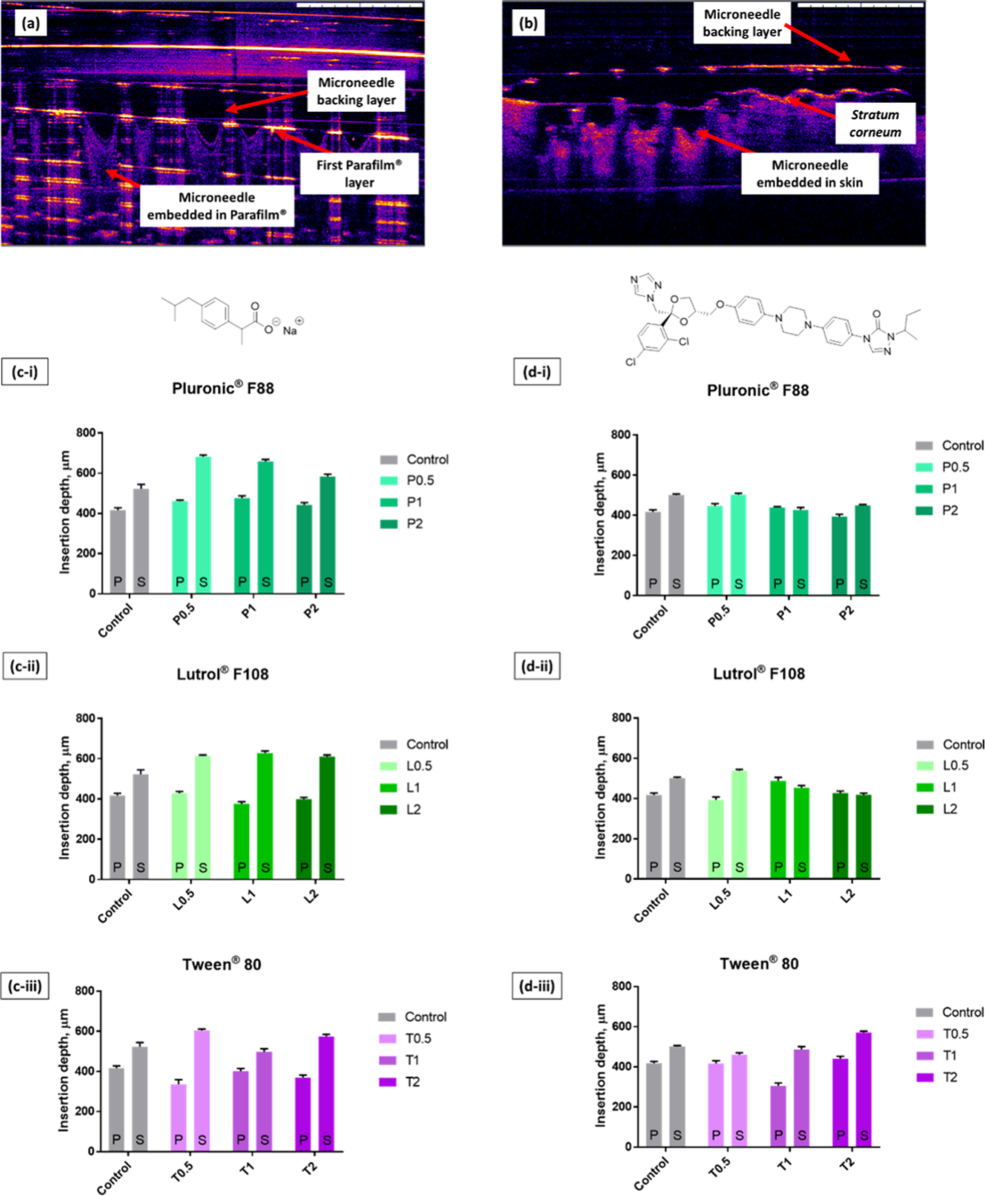
Microneedle penetration into (a) Parafilm and (b) full-thickness neonatal porcine skin as monitored *via* optical coherence tomography. Comparison of the insertion depth of dissolving MAPs during Parafilm (P) and skin (S) insertion for dissolving MAPs loaded with (c) ibuprofen sodium and (d) itraconazole (mean + SD, *n* = 3).

To further evaluate the penetration profiles of the MAP formulations developed, the Parafilm insertion studies were complemented with *ex vivo* neonatal porcine skin insertion experiments. Upon application into stacks of Parafilm and *ex vivo* skin, the penetration depth of the MAPs was imaged and measured using optical coherence tomography (OCT). OCT was utilized as the method to measure MAP penetration depth following skin insertion over histological sectioning, as this technique overcomes the issues associated with altering the skin structure during the cryo-sectioning step that could lead to erroneous estimation of the microneedle penetration depth.^61^ Examples of OCT images obtained from the analysis of microneedle penetration *in situ* into Parafilm and skin are shown in Figure 10.

It can be seen from Figure 10 that the overall microneedle penetration depth was considerably less than the overall microneedle length, which is approximately 850 μm. This is attributed to the inherent viscoelastic properties of both Parafilm M and skin, which resist microneedle penetration, resulting in incomplete microneedle insertion.^62^ In the control formulation, consisting of either ibuprofen sodium or itraconazole with PVA and PVP, we observed that the microneedle insertion into *ex vivo* skin was significantly deeper (*p* < 0.05) than that into Parafilm. This observation might be attributed to the mechanical properties of neonatal porcine skin and the Parafilm stack. Also, this was attributed to the absence of water in the Parafilm insertion test, which prevented the polymeric microneedles from expanding resulting in a shallower insertion depth into the Parafilm stack relative to the *ex vivo* neonatal porcine skin. In addition, it can be seen from Figure 10c that the addition of surfactant significantly improved the insertion profile (*p* < 0.05) for ibuprofen sodium-loaded dissolving MAPs relative to the control formulation.

This might be attributed to the role of surfactant as a wetting agent that lowers the surface tension at the solid–liquid interface, thus promoting the spread and penetration of liquid into solid polymeric matrixes.^63,64^ In this instance, the presence of surfactant along the microneedles helps to promote the wetting and spreading of the dermal interstitial fluid along and into the microneedle polymeric matrix. The wetting of the interstitial fluid along the microneedle surface leads to a phenomenon known as boundary lubrication, which mitigates the friction experienced by the polymeric surface with another solid surface, thus enabling deeper microneedle insertion under the same applied force.^65^

In contrast, it can be seen that the addition of surfactant into itraconazole-loaded dissolving MAPs did not enhance microneedle insertion into the skin to the same extent as ibuprofen sodium-loaded dissolving MAPs. It is postulated that the hydrophobic nature of itraconazole mitigated the spreading of the dermal interstitial fluid along the microneedle surface and into the microneedle polymeric matrix. This ultimately led to less boundary lubrication upon application leading to shallower microneedle penetration into *ex vivo* skin. Nevertheless, it was observed that the addition of higher concentrations of Tween 80 (1.0 and 2.0% w/w of surfactant solutions) managed to improve the insertion profile of itraconazole-loaded dissolving MAPs.

### 3.6 Drug Loading, *In Situ* Dissolution Study, and Delivery Efficiency in *Ex Vivo* Neonatal Porcine Skin

**Figure 11:**
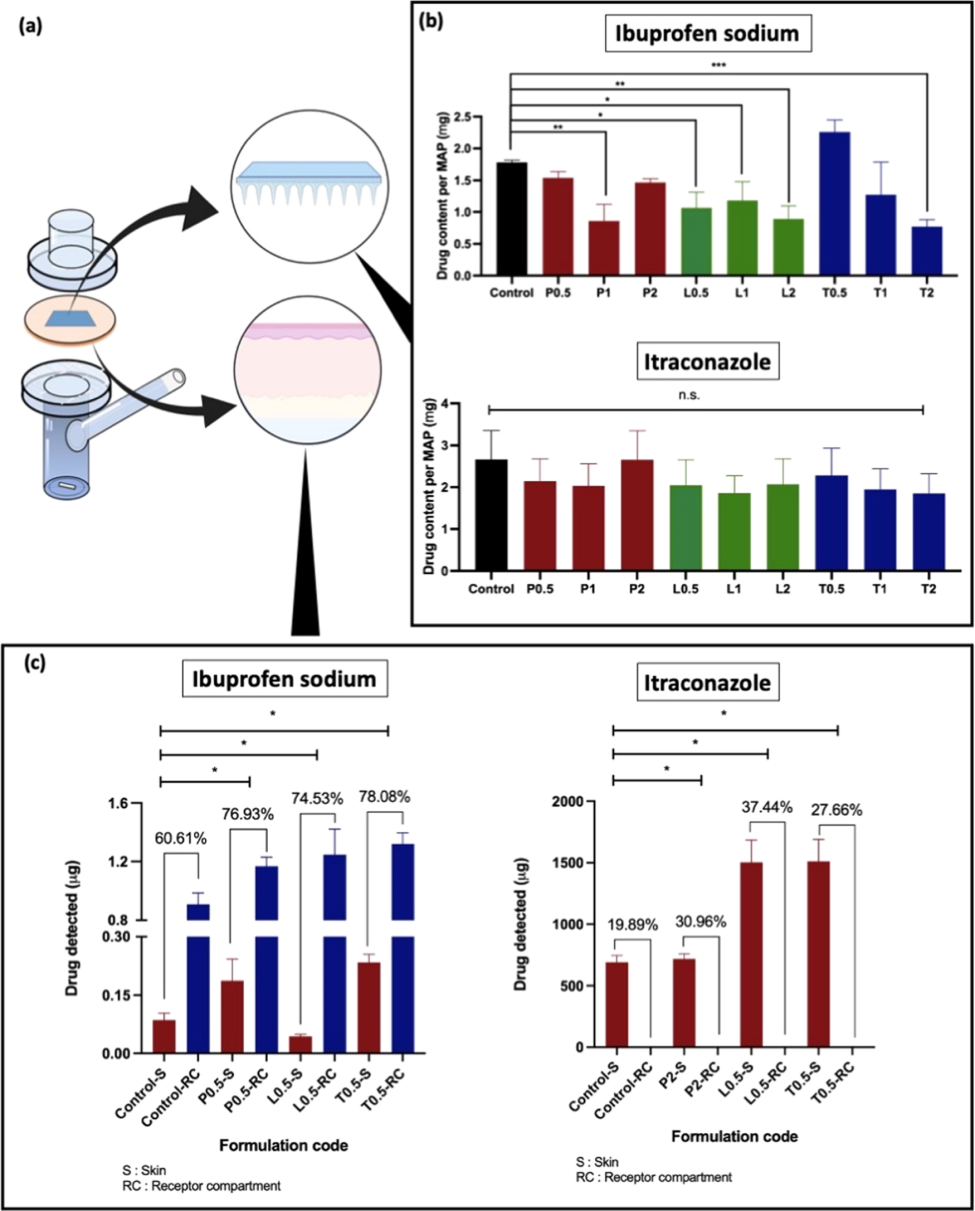
(a) Schematic illustration of the modified Franz cell setup. (b) Comparison of drug content per MAP for ibuprofen sodium and itraconazole (means + SD, *n* = 3). (c) Results of the *ex vivo* skin deposition study and drug delivery efficiency of ibuprofen sodium and itraconazole from dissolving MAPs in full-thickness neonatal porcine skin after 24 h of application (means + SD, *n* = 3).

In the present work, the drug loading of ibuprofen sodium and itraconazole into polymeric microneedle patches with the incorporation of different surfactants of varying concentrations was evaluated. It can be seen from Figure 11 that the addition of surfactant may, in some instances, impact the amount of drugs that can be loaded into the MAPs. In the case of ibuprofen sodium, it can be seen that the addition of surfactant resulted in a reduction in the amount of drug loaded per microneedle patch. In contrast, the incorporation of surfactant did not have any impact on the amount of itraconazole that could be loaded into the microneedle patches.

The lower ibuprofen sodium loading for the respective dissolving MAPs upon incorporating surfactant may be attributed to the increase in viscosity of the casted polymer blend with the incorporation of surfactant. It has been shown by several researchers that the addition of low-concentration surfactants indeed results in an increase in the polymer blend viscosity.^66,67^ In preparing the patches, the microneedle layers were formed through the micromolding technique, which involved casting and subjecting the drug–polymer blend under positive pressure to push the blend into the poly(dimethylsiloxane) mold. The increase in the polymer blend viscosity upon incorporating surfactant may result in an increase in resistance in filling these microneedle molds, which ultimately results in reduced drug loading. However, such an observation was only apparent for the model hydrophilic drug ibuprofen sodium but not for the model hydrophobic drug itraconazole.

Upon quantifying the drug loading for the respective MAPs, a skin deposition study using full-thickness neonatal porcine skin was conducted. The skin deposition study was conducted using a Franz cell setup as illustrated in Figure 11a. Microneedle formulations loaded with either ibuprofen sodium or itraconazole without any surfactant were selected as the control formulation. Based on the microneedle characterization, only microneedle formulations with the highest drug loading for respective drug molecules were evaluated in the skin deposition study. It can be seen from Figure 11c that for the control formulations, ibuprofen sodium displayed a higher delivery efficiency (60.61%) relative to itraconazole (19.89%). Such a difference in delivery efficiency may be attributed to the higher water solubility of ibuprofen sodium relative to itraconazole. In addition, the higher water solubility for ibuprofen sodium resulted in the permeation of the drug across the skin and into the receptor compartment. In contrast, due to the poor aqueous solubility of itraconazole (1–4 ng/mL), the drug was only able to be deposited into the skin tissue with no detectable levels within the receiver compartment.

However, it was apparent that the addition of surfactant for both model drugs, ibuprofen sodium and itraconazole, resulted in an improvement in the overall delivery efficiency of the microneedle formulations. With respect to ibuprofen sodium, the control formulation only displayed a delivery efficiency of 60.61%. However, the addition of surfactant resulted in an increase in the drug delivery efficiency of up to ≈75% into and across the skin. It was apparent that the dissolving microneedle patch that incorporated Tween 80 displayed the highest delivery efficiency of ibuprofen sodium (78%) out of all of the formulations evaluated for the skin deposition study. In addition, for all of the formulations evaluated, most of the ibuprofen sodium was delivered across the skin and was present in the receptor compartment as shown in Figure 11c. In addition, formulations that were loaded with surfactants resulted in a higher transdermal delivery of ibuprofen sodium into the receptor compartment relative to the control formulation. From a clinical standpoint, it can be postulated that the incorporation of surfactants such Tween 80 may be a viable strategy to improve the transdermal delivery efficiency of highly water-soluble drugs, such as ibuprofen sodium, across the skin and into the systemic circulation. On the other hand, it is also clear that the addition of surfactant did improve the delivery efficiency of itraconazole, as shown in Figure 11c. However, such an enhancement in delivery efficiency did not result in any improvement in the amount of drug delivered across the skin as there were no detectable levels of itraconazole within the receiver compartment. It can be seen that dissolving microneedle patches that incorporated Lutrol F108 displayed the highest delivery efficiency of itraconazole into the skin (37.44%) out of all of the formulations evaluated. This result suggests that for hydrophobic drugs that are intended for intradermal administration such as those indicated for localized therapy, the incorporation of nonanionic surfactants may be a viable strategy to improve the delivery efficiency and the amount of drug delivered into the skin.

In addition, it can also be seen from Figure 11a that the improvement in delivery efficiency is mainly attributed to the presence of surfactant and not due to an improvement in the drug loading content. This is especially true for itraconazole as there are no significant (*p* > 0.05) changes in drug loading with the addition of surfactants, but we observed an increase in delivery efficiency into the skin. In the case of ibuprofen sodium, indeed the inclusion of surfactants did impact the drug loading of some formulations. However, the formulations that were evaluated in the *in vitro* permeation study contain similar (*p* > 0.05) loadings of ibuprofen sodium, with the exception of L0.5 which contains a lower loading. Therefore, such an enhancement in delivery efficiency observed in Figure 11c was mainly attributed to the presence of surfactants.

**Figure 12:**
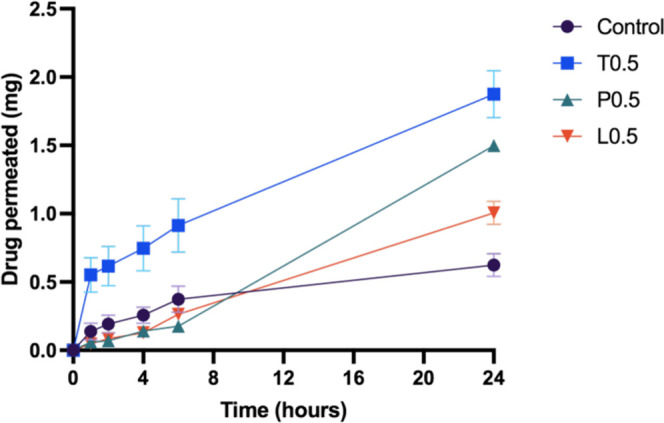
Results of the *in vitro* permeation study and drug delivery efficiency of ibuprofen sodium from dissolving MAPs in full-thickness neonatal porcine skin over 24 h (means + SD, *n* = 4).

To evaluate how the nonionic surfactant affects the release profile of dissolving microneedles over time, we conducted a further *in vitro* permeation experiment using full-thickness neonatal porcine skin. In this study, the receptor fluid was sampled over the course of 24 h in an attempt to elucidate the release profile of the model drug, ibuprofen sodium, into the receptor chamber of Franz cells from MAP formulations containing different types of nonionic surfactants. Ibuprofen sodium was chosen as the model drug as it is highly hydrophilic and is capable of traversing the skin and into the receptor chamber. Attempts have been made to quantify itraconazole permeation across the skin. However, the poor aqueous solubility of this antifungal agent prevents the permeation of itraconazole, at a detectable level, into the receptor chamber of the Franz cells. From Figure 12, we observed that the inclusion of surfactants such as Lutrol F108 and Pluronic F88 did not result in any improvement in the release profile of the drug during the first 6 h of the permeation study. Nevertheless, we observed a significantly higher level of ibuprofen sodium permeation at 24 h (*p* < 0.05) for MAPs loaded with Lutrol F108 and Pluronic F88 relative to the control. In contrast, the inclusion of Tween 80 resulted in both a more rapid release of ibuprofen sodium (*p* < 0.05) within the first 6 h of the study and an overall higher drug release at 24 h (*p* < 0.05) relative to the control and other formulations evaluated, which is analogous to drug delivery efficiency (data shown in Figure 11c).

**Figure 13:**
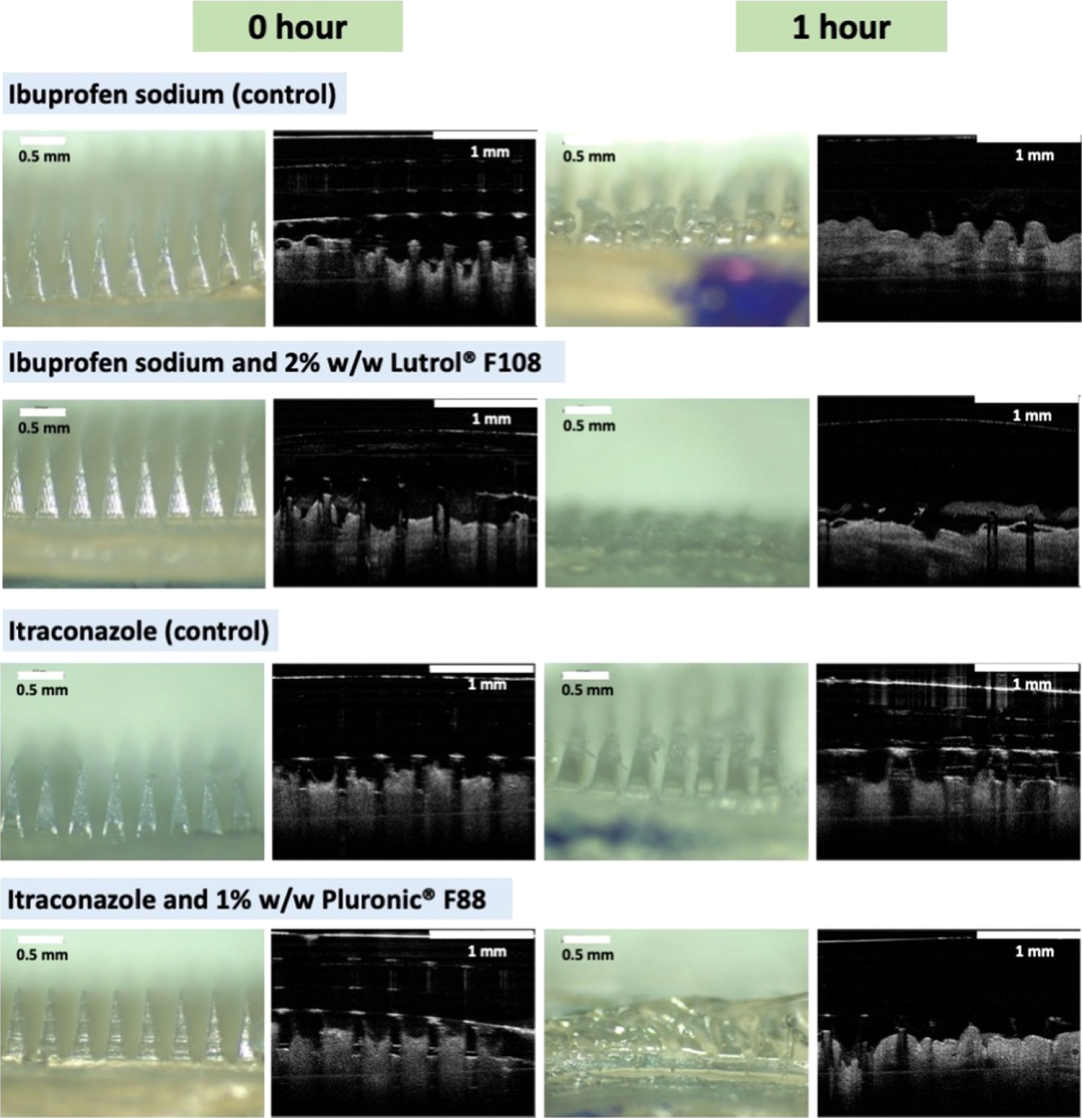
Digital and OCT images of needle dissolution at 0 and 1 h following insertion into and removal from excised neonatal porcine skin *ex vivo*.

It has been reported in the literature that the incorporation of surfactants into pharmaceutical formulations is one of the strategies to improve the drug delivery efficiency of poorly water-soluble drugs. Such improvement in delivery efficiency through the use of surfactant may be attributed to the effect of surfactant in enhancing the overall hydrophilicity of the formulation, in this case, the microneedle layers.^68^ The incorporation of surfactant into a pharmaceutical formulation improves the surface wettability of the formulation, particularly polymeric films, upon encountering an aqueous milieu.^69^ In this instance, the insertion of microneedles into the skin exposes the surface of the PVP/PVA MAP to the water-rich dermis. The presence of surfactant, such as Pluronic F88, enhances the microneedle surface wettability upon insertion into the dermis. This is subsequently accompanied by an increase in the water ingress into the polymeric matrix of the microneedle layer.^70^ Enhancement of the rate of water penetration into the polymeric microneedle layer results in an increase in the overall swelling and the dissolution rate of PVP and PVA within the dermis that culminates in the release of itraconazole into the skin. In the absence of surfactant, the microneedle layer is very hydrophobic due to the presence of itraconazole, which hinders the rate of PVP/PVA dissolution within the skin resulting in incomplete drug deposition into the skin. This may also serve as an explanation for the *in situ* skin dissolution studies where microneedles that incorporated surfactants were capable of dissolving within an hour relative to the control formulation, as evident in Figure 13. The results from Figures 11 and 13 collectively suggest that the incorporation of surfactants such as Lutrol F108 did not only improve the delivery efficiency of the model drugs ibuprofen sodium and itraconazole but also enabled the complete dissolution of the needle layer within 1 h relative to the control formulation. Overall, this skin deposition study illustrated that the incorporation of surfactant was a viable strategy to improve the intradermal delivery efficiency of poorly water-soluble drugs into the skin while enabling rapid dissolution of the microneedle layers. Indeed, it can be seen that the addition of nonionic surfactant provides a means of enhancing the properties and performance of dissolving microneedles. Nevertheless, the current work only explored a limited range of pharmaceutical polymers, albeit the most common (PVA and PVP), used in fabricating dissolving microneedles. Indeed, caution should be exercised when extrapolating such results to other ranges of polymers. Therefore, future work is warranted to further explore the effect of different polymer combinations in tandem with different surfactants on the properties of dissolving microneedles.

In the process of fabricating dissolving MAPs *via* micromolding, the selection and incorporation of additives play a pivotal role in the overall properties and performance of dissolving MAPs. For example, the addition of inert excipients may in some instances improve the physical characteristics of MAPs such as tensile strength. However, the addition of inert excipients will not, to any noticeable extent, affect the delivery efficiency of MAPs. For instance, previous works by other researchers such as Yu et al.^71^ have explored the inclusion of physiologically inorganic excipients such as calcium sulfate hemihydrate into gelatin-based dissolving MAPs. The addition of the excipient resulted in an improvement in the mechanical strength of the needles although such excipients did not play any additional role in improving the delivery of the payload into the skin. In addition, earlier studies in dissolving MAPs have explored the inclusion of inert excipients such as glycerol and low-molecular-weight PEG as a plasticizer to improve MAP flexibility while mitigating microneedle fracture during the demolding stage.^72,73^ However, more recent studies in the microneedle field such as those conducted by Ramirez et al.^74^ have begun exploring functionally active excipients, such as magnesium microparticles, which form mini-pneumatic-pumps upon contact with the dermal interstitial fluid resulting in the improvement of the MAP dissolution rate and the dermal distribution of the drug upon application while still providing some degree of improvement in terms of the physical properties of MAPs. Therefore, we believe the use of a functionally active excipient such as surfactants, which is more commonly used in drug delivery, may enhance the physical properties of MAP and the delivery efficiency of the overall formulation as shown in the current work. Besides this, the use of surfactant as an additive in dissolving MAP fabrication may be viewed as an additional approach in refining and designing dissolving MAPs.

## 4 Conclusions

In conclusion, the current work highlights the fabrication, characterization, and evaluation of a series of dissolving polymeric MAPs consisting of different nonionic surfactants, Tween 80, Lutrol F108, and Pluronic F88, of varying concentrations. Dynamic light scattering indicated that the incorporation of Pluronic F88 and Lutrol F108 resulted in a 10-fold reduction in the ibuprofen sodium drug particle size from 5400 to 500 nm, while the addition of Tween 80 solution at concentrations of 1.0 and 2.0% w/w resulted in around a 500-fold decrease in the particle size down to 10 nm. The fabricated MAPs displayed good microneedle architecture and possessed sharp microneedle tips as evidenced by microscopy. In addition, all of the dissolving MAPs that incorporated surfactant displayed a lower reduction in microneedle height (≈10%) relative to the control formulation (≈20%) when subjected to a compressive force of 32 N. Furthering this, the insertion study showed that the MAPs were capable of breaching an *in vitro* skin simulant (Parafilm M) and *ex vivo* skin, which was evidenced *via* OCT analysis. The insertion study using excised *ex vivo* neonatal porcine skin showed that incorporation of surfactant into ibuprofen sodium-loaded dissolving MAPs improved the insertion depth of MAPs from 400 μm down to 600 μm. However, such enhancement was not apparent when the MAPs were loaded with the model hydrophobic drug itraconazole. On the other hand, DSC analysis indicated that the model drugs, ibuprofen sodium and itraconazole, that were loaded into these dissolving MAPs were present in a low crystalline state and that the addition of surfactant did not induce any drug recrystallization within the formulation. Lastly, the skin deposition study highlighted that the incorporation of surfactant resulted in a significant enhancement in the delivery efficiency of both model drugs, ibuprofen sodium and itraconazole. With respect to ibuprofen sodium, the addition of surfactant enhanced the amount of drug delivered from 60.61% up to ≈75% with a majority of the drug being delivered across the skin and into the receptor compartment. On the other hand, when surfactants were added into MAPs loaded with the model hydrophobic drug itraconazole, we also observed enhancement in intradermal delivery efficiency from 20% up to 30%, although this did not improve the delivery of the drug across the skin. Collectively, the current work highlights that the incorporation of nonionic surfactants into dissolving MAPs could be an alternative formulation strategy that could be explored by formulators to enhance not only the mechanical resistance and insertion profile of dissolving polymeric MAPs but also the delivery efficiency of the delivered therapeutics.
